# Requirement of Mouse BCCIP for Neural Development and Progenitor Proliferation

**DOI:** 10.1371/journal.pone.0030638

**Published:** 2012-01-24

**Authors:** Yi-Yuan Huang, Huimei Lu, Stephany Liu, Roberto Droz-Rosario, Zhiyuan Shen

**Affiliations:** The Cancer Institute of New Jersey, Department of Radiation Oncology, Robert Wood Johnson Medical School, New Brunswick, New Jersey, United States of America; National Cancer Institute, United States of America

## Abstract

Multiple DNA repair pathways are involved in the orderly development of neural systems at distinct stages. The homologous recombination (HR) pathway is required to resolve stalled replication forks and critical for the proliferation of progenitor cells during neural development. BCCIP is a BRCA2 and CDKN1A interacting protein implicated in HR and inhibition of DNA replication stress. In this study, we determined the role of BCCIP in neural development using a conditional BCCIP knock-down mouse model. BCCIP deficiency impaired embryonic and postnatal neural development, causing severe ataxia, cerebral and cerebellar defects, and microcephaly. These development defects are associated with spontaneous DNA damage and subsequent cell death in the proliferative cell populations of the neural system during embryogenesis. With *in vitro* neural spheroid cultures, BCCIP deficiency impaired neural progenitor's self-renewal capability, and spontaneously activated p53. These data suggest that BCCIP and its anti-replication stress functions are essential for normal neural development by maintaining an orderly proliferation of neural progenitors.

## Introduction

Deficiencies of distinct DNA repair pathways affect specific cell populations at different developmental stages, thus cause various degrees of neural degeneration and developmental disorders [1]–[3]. Early in development, when proliferation of progenitor cells is critical for neurogenesis, the homologous recombination (HR) machinery is crucial to ensure proper completion of DNA replication with high fidelity, thus an orderly development. Among post-mitotic cell populations, the non-homologous end joining (NHEJ) pathway may be critical to maintain genome stability and orderly differentiation. In the mature neural tissues when there is little cell proliferation or differentiation, accumulation of oxidative damage is a major obstacle for normal function. In this case, DNA repair pathways relieving oxidative damage (such as excision repair) are critical to prevent neurodegenerative syndromes [4], [5].

BCCIP was originally known as a BRCA2 and CDKN1A (Cip1/waf1/p21) interacting protein, and it plays a wide range of regulatory roles in the HR pathway, cytokinesis, and cell cycle regulation [6]–[16]. In addition, BCCIP may have functions in neurite growth, cell mobility, and nuclear and cytoplasmic shuttling [17]–[19]. Because the HR machinery is critical for neural development, we hypothesized that BCCIP plays a role in neural development. In this study, we found that BCCIP deficiency causes proliferation arrest among progenitor cells, leading to severe neurogenesis defects, including: microcephaly, ataxia, cerebral and cerebellar development disorders, and growth retardation. These observations suggest a key role of BCCIP in neural development.

## Results

### Establishment of a glial fibrillary acidic protein promoter driven (*GFAP*) Cre-mediated conditional BCCIP knockdown mice

**Figure 1:**
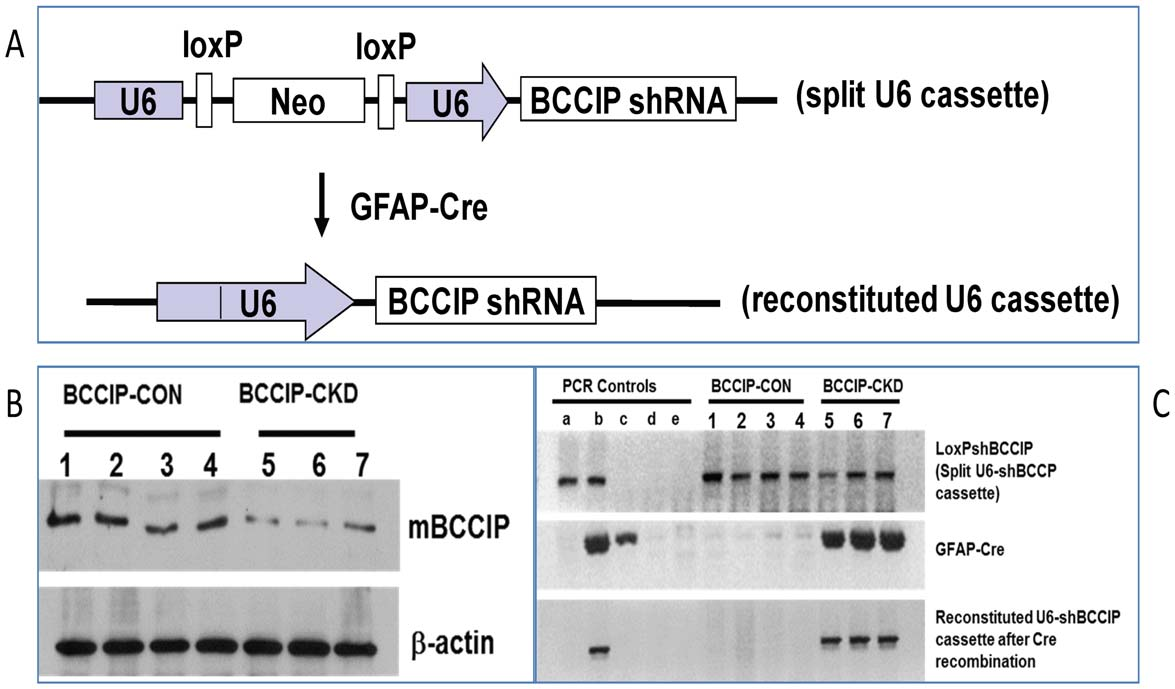
GFAP-Cre mediated conditional knockdown of BCCIP. (**A**) shows the strategy of LoxP-Cre mediated conditional expression of shRNA against mouse BCCIP gene. The U6 promoter is split and inactivated by the insertion of a LoxP-neo-LoxP fragment (upper panel). Upon expression of Cre-recombinase, the deletion of the Neo cassette between the LoxP sites reconstitutes a functional U6 promoter that drives the expression of the shRNA. (**B**) BCCIP protein levels from a representative litter of 7 mice resulting from breeding between *LoxPshBCCIP^+/+^* and *GFAP-Cre* ^+/−^. The brain tissues from 4 *BCCIP-CON* and 3 *BCCIP-CKD* mice at age P1 were used for DNA and protein extractions. Shown are Western blots of the brain protein extracts, and b-actin (loading control). (**C**) genotyping of the same litter of mice as panel B. The upper two panels are results from tail DNA to verify the presence of the split U6 promoter *LoxPshBCCIP* and the *GFAP-Cre* cassettes. The bottom panel is results verifying the reconstituted U6-shBCCIP cassette using DNA from the brain tissue of the mice. Five lanes (a, b, c, d, and e) of controls are also shown: a - DNA from the BCCIP-CON mice (*LoxPshBCCIP* ^+/−^;*GFAPCre* ^−/−^) ; b – DNA from a *BCCIP-CKD* mouse (*LoxPshBCCIP* ^+/−^;*GFAP-Cre* ^+/−^); c – DNA from a *GFAPCre* mouse; *d -* DNA from wild type mouse; and e - water as a negative PCR control. As shown here, all 7 (No. 1-7) littermates contain the original split U6 cassette in their tail DNA. But only the littermates (No. 5-7) with the *GFAPCre* cassette have reconstituted U6-shBCCIP cassette in the DNA extracted from brain tissues at P1.

In humans, two major alternative splicing products of BCCIP (BCCIPα and BCCIPβ are expressed, with BCCIPβ being the main isoform. However, it appears that mouse tissues expressed only the BCCIPβ isoform. To investigate the functions of BCCIP in neurogenesis, we used a conditional BCCIP knockdown transgenic mouse line [16], designated *LoxPshBCCIP.* As briefly illustrated in Figure 1A, in this model, the expression of Cre-recombinase enables the expression of a short hairpin RNA against mouse BCCIP (shBCCIP). We crossed the *LoxPshBCCIP* mice with heterozygous *GFAP-Cre^+/−^* transgenic mice, which express the Cre recombinase under the control of the human glial fibrillary acidic protein promoter (*GFAP*) [20]. The GFAP promoter becomes active at around embryonic day 13.5 (E13.5) in multi-potential stem cells in multiple regions during embryogenesis, including neuron and glial progenitors [20]. However, in adult mice the GFAP-Cre recombinase is expressed mainly in glial cells [20]. After crossing *LoxPshBCCIP^+/+^* with *GFAP-Cre^+/−^* transgenic mice, we obtained 67 mice with a 32∶35 ratio between *LoxPshBCCIP^+/−^;GFAPCre^−/−^* and *LoxPshBCCIP^+/−^;GFAPCre^+/−^* (hereafter referred as *BCCIP-CON* and *BCCIP-CKD*, respectively) among 7 liters. Effective BCCIP knockdown and Cre-mediated reconstitution of the functional U6 promoter in *BCCIP-CKD* mice was verified by Western blots of brain protein extracts (Figures 1B) and by genotyping on genomic DNA from brain extracts (Figure 1C).

### Growth retardation, ataxia, and balance disorder of *BCCIP-CKD* mice

**Figure 2:**
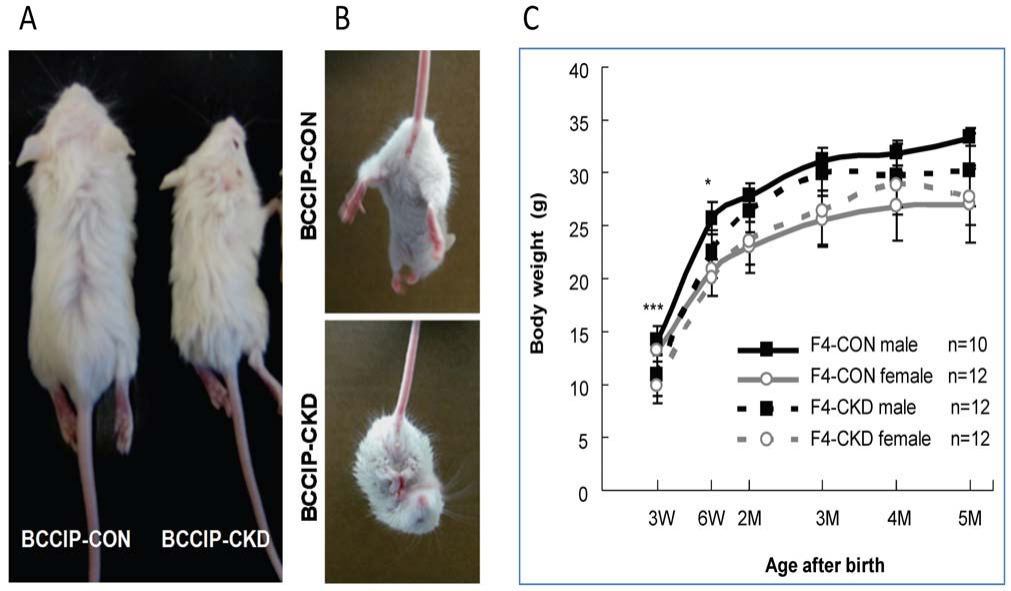
Growth retardation, ataxia, and motor reflex defects in *BCCIP-CKD* mice. (**A**) shows a representative pair of littermates at age P21. (**B**) shows a typical motor reflex response by control (top panel) and BCCIP knockdown mice (bottom panel) at age P21. (**C**) The body weight of control (*BCCIP-CON*) and knockdown (*BCCIP-CKD*) mice were measured at various ages. Shown are averages (+/− standard deviation) of male and female mice at different ages. The n-values represent the numbers of the mice at each age. *: p<0.05; **: P<0.01; ***: P<0.001.

The *BCCIP-CKD* mice had similar body weight as their *BCCIP-CON* littermates at birth. However, postnatal growth delay of *BCCIP-CKD* mice became evident in the first few weeks. By weaning (age P21), the body weight of *BCCIP-CKD* mice reduced to ∼70% of the *BCCIP-CON* mice (Figures 2A). After age P14, all *BCCIP-CKD* mice displayed various degrees of walking disorders and unkempt coats, which are likely caused by poor motor coordination. All *BCCIP-CKD* mice show balance disorder, tremors, and akinesis. About 60% *BCCIP-CKD* mice had severe ataxia (see Supplement Movie S1), and could not pass the balance beam test at age P21. Because those symptoms implicate motor neuron defects, we assessed motor reflex by the hind-limb extension test. In this test, an extension reflex of the hind-limbs (Figure 2B, top panel) can be observed when a normal mouse is suspended by its tail. When *BCCIP-CKD* mice were suspended by its tail, 80% of *BCCIP-CKD* mice displayed hind-limb retraction in a crossed posture (Figure 2B, bottom panel).

The *BCCIP-CKD* mice did not show a significantly shorter lifespan compared with *BCCIP-CON* mice. While most of the ataxic symptoms among *BCCIP-CKD* mice persist between the ages of P14 and P28, which is the time period critical for the development of motor neurons and dendritic growth in the cerebellum, we noticed a gradual relief of the severe ataxic symptoms after age P42. By age P56, the severe ataxic symptoms were not noticeable, although the *BCCIP-CKD* still failed the balance beam test throughout their life. Coincidental with the relief of ataxia symptom around age p28, the *BCCIP-CKD* mice began to gain weight. After P56, the *BCCIP-CKD* mice body weight was similar to that of the *BCCIP-CON* mice (Figure 2C).

**Figure 3:**
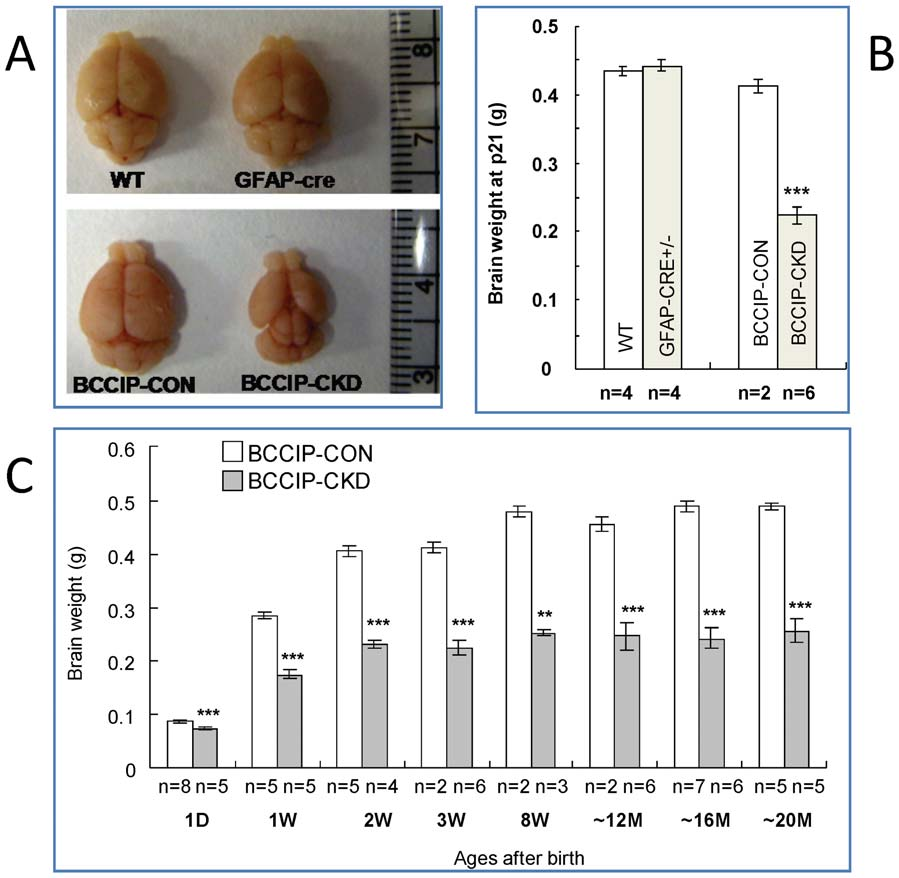
*GFAP-Cre* mediated conditional knockdown of BCCIP causes microcephaly. (**A**) a representative set of brains from wild type (WT), *GFAP-Cre*, *BCCIP-CON*, and *BCCIP-CKD* mice at P21, illustrating the reduced cerebrum and cerebellum in the *BCCIP-CKD* mice. (**B**) the brain weight of wild type (WT), *GFAP-Cre*, *BCCIP-CON* and *BCCIP-CKD* mice at age P21. (**C)** brain weight of *BCCIP-CON* (white bar) and *BCCIP-CKD* (gray bar) mice at various ages, ranging from day 1 (1D) to approximately 18 months. Asterisks indicate the statistic significance between *BCCIP-CON* and *BCCIP-CKD* of the same age (*: P<0.05; **: P<0.01; ***: P<0.001). The “n” values indicate the number of mice measured at the time point. D: day; W: week; and M: month.

To elucidate the cause of these abnormalities among *BCCIP-CKD* mice, we surveyed gross brain development. At postnatal day 21 (P21), the brain weight of *BCCIP-CKD* mice was significantly reduced compared to littermate *BCCIP-CON* mice, *GFAP-Cre* mice, and wild type mice (Figures 3A and 3B). The reduction in brain size of *BCCIP-CKD* mice was observed as early as postnatal day 1 (P1), and throughout adulthood it remained at approximately 50% of the littermate control brain after postnatal day 14 (P14) (Figure 3C). In contrast, a relatively mild difference was observed on body weight (Figure 2C). The affected regions in *BCCIP-CKD* brain include both cerebrum and cerebellum (Figure 3A).

### Widespread defects in neurogenesis among BCCIP deficient mice

**Figure 4:**
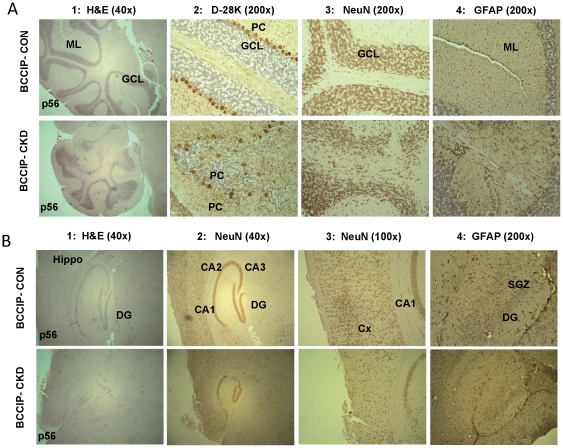
Histological analysis of cerebellum and sagittal brain sections of *BCCIP-CON* and *BCCIP-CKD* mice. (**A**) Histology of cerebellum of *BCCIP-CON* (top row) and *BCCIP-CKD* (bottom row) mice at age P56. Column 1 shows Hematoxylin and Eosin (H&E) staining of cerebellum at magnification of 40×. Columns 2–4 show the immuno-histochemical staining for Calbindin (D-28K) that is specific for Purkinje cells (brown color in column 2), NeuN that is specific for neurons (brown color in column 3), and for GFAP is specific for glial cells (brown color in column 4). ML: molecular layers; GCL: granule cell layer; PC: Purkinje cells. (**B**) Histology of cerebrum of *BCCIP-CON* and *BCCIP-CKD* mice at age P56. Column 1 shows the H&E staining at 40× magnification. Columns 2 and 3 show the reduced neuron density in BCCIP-CKD mice as visualized by anti-NeuN staining. Columns 4 shows reduced density of glial cells (stained with anti-GFAP) in the *BCCIP-CKD* mice. Hippo: hippocampus; DG: dentate gyrus; CA1, CA2 and CA3: pyramidal cell layer of the hippocampus; Cx: cortex; SGZ: subgranular zone.

Between embryonic day 10 (E10) to postnatal day 2 (P2), various regions of the mouse brain, including the cerebral cortex, the midbrain, and the cerebellum, undergo rapid expansion [21]. The hGFAP promoter actively drives expression of the Cre recombinase at around E13.5 in multi-potential neural stem cell in the afore-mentioned regions [20]. To understand the cause of microcephaly, we performed histological analyses on cerebellum and cerebrum regions. In the cerebellum of *BCCIP-CKD* mice, we found an agenesis in foliation and lobule structure (Figure 4A). The B*CCIP-CKD* cerebellum displayed reduced granule cell number, disrupted granule cell layers, and abnormal lining pattern of Purkinje cells (Figure 4A). Furthermore, reduced number of Bergmann glial cells was also observed in *BCCIP-CKD* cerebellum (Figure 4A). In the cerebrum of *BCCIP-CKD* mice, despite that the cortical laminar structure and the gross structures of hippocampus were largely preserved, there was a significant reduction of neuron cell density in *BCCIP-CKD* cortex (Figure 4B). These observations suggest that BCCIP defect impairs the development of both cerebellum and the cerebrum.

### Increased apoptosis and reduced proliferation in the progenitor rich embryonic external germinal layer of cerebellum and ventricular zone of cortex of the *BCCIP-CKD* mice

The gross evaluation of the brains BCCIP deficient mice (Figure 3A) revealed a smaller size in both the cerebellum and the cerebrum regions. Reduced neurogenesis and microcephaly may be a consequence of an increased rate of apoptosis or reduced neural progenitor proliferative capacity. We investigated the effect of BCCIP deficiency on the progenitor cell populations that contribute to the development of cerebellum and cerebrum.

**Figure 5:**
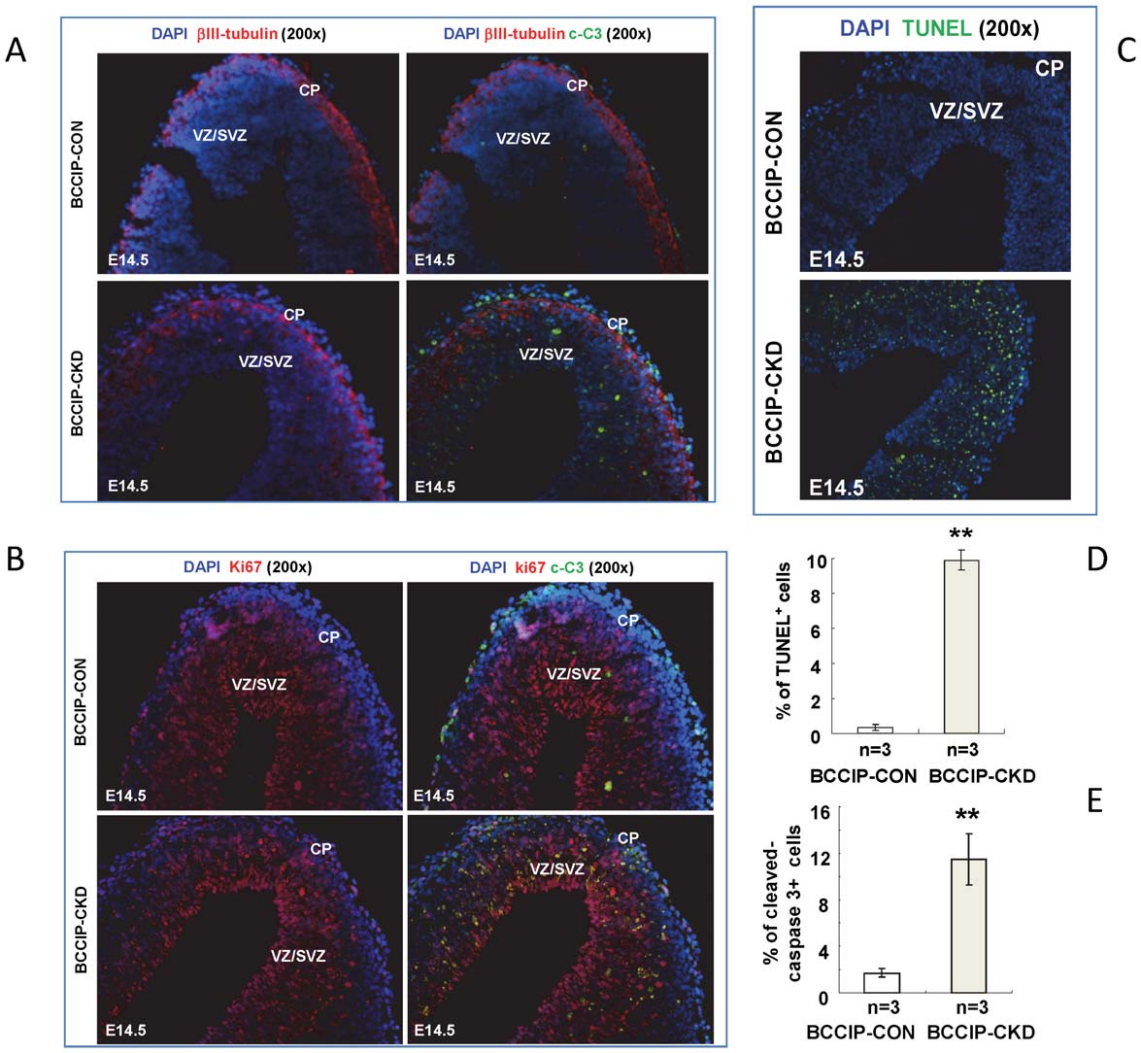
BCCIP knock-down increased apoptosis in the mitotic proliferating region of *BCCIP-CKD* neocortices. (A) βIII-tubulin staining identifies the differentiating neural cell populations. Analysis of apoptosis was done by cleaved-caspase 3 (c-C3) staining in E14.5 using *BCCIP-CON* and *BCCIP-CKD* embryos. The merged composites are overlay of βIII-tubulin and DPAI staining. (**B**) Ki67 staining identifies the proliferating mitotic region. Analysis of apoptosis was done by cleaved-caspase 3 (c-C3) staining in E14.5 using *BCCIP-CON* and *BCCIP-CKD* embryos. (**C**) Apoptosis was analyzed by TUNEL assay in E14.5 using *BCCIP-CON* and *BCCIP-CKD* embryos. (**D**) Quantification of TUNEL staining. The amount of apoptosis was quantified in the VZ/SVZ of E14.5 embryos. (**E**) Quantification of cleaved-caspase 3 staining. The amount of apoptosis was quantified in the VZ/SVZ of E14.5 embryos. CP: cortical plate. VZ/SVZ: ventricular zone/subventricular zone. White bars: *BCCIP-CON*; Gray bars: *BCCIP-CKD*. The merged composites are overlay of Ki67 and DAPI staining. CP: cortical plate. VZ/SVZ: ventricular zone/subventricular zone. *: P<0.05; **: P<0.01; ***: P<0.001.

Mouse cortical neurogenesis occurs mostly from embryonic days 11 to 19 (E11–E19). It derives from a germinal layer of the dorsal telencephalon at E11 and begins to form during mouse mid-embryogenesis [21], [22]. To determine the cell type affected by BCCIP down-regulation in neocortices, we stained Ki67 and βIII-tubulin to label proliferating neural progenitor cells and newly differentiated neurons at embryonic day 14.5 (E14.5). At this stage the cortical neural progenitors including radial glial cells and short neural precursors proliferate in the ventricular zone (VZ), while intermediate progenitor cells (derived from radial glial cells) divide in the subventricular zone (SVZ). As shown in Figure 5, these progenitor layers displayed a decreased breadth on the BCCIP deficient mice. By contrast, the width of βIII-tubulin-positive zone (corresponding to the differentiated region) was not affected in BCCIP deficient mice (Figure 5A). The boundary of differentiated βIII-tubulin-positive zone in *BCCIP-CKD* mice was not as well defined as in *BCCIP-CON* mice (Figure 5A). Increased apoptosis in both VZ and SVZ occur mainly in Ki67 positive region but âIII-negative layer (Figure 5B) as seen in immunoflourescent co-staining for cleaved Caspase-3 (a marker for apoptosis) with βIII-tubulin (Figure 5A) and Ki67 (Figures 5B and 5E), and TUNEL assay (Figure 5C and 5D). Little apoptosis was observed in βIII-tubulin-positive postmitotic region (Figure 5A). These observations indicate that defects in neocortical development in *BCCIP-CKD* embryos likely resulted from reduction of proliferative capacity and increased apoptosis of progenitors.

**Figure 6:**
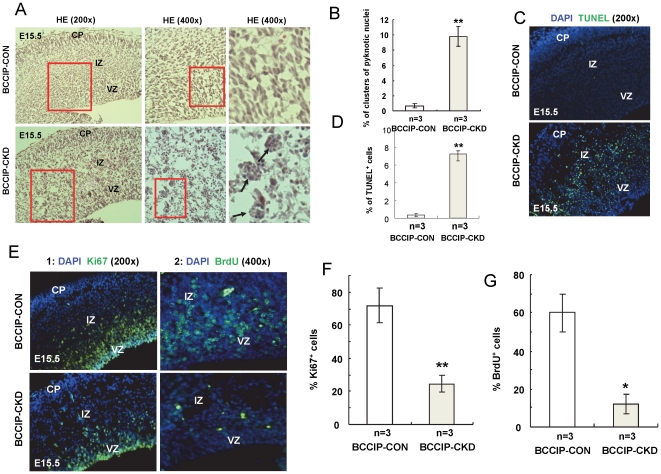
BCCIP knock-down causes apoptotic cell death and reduction of cell proliferation capacity in the neocortices progenitor cells. The brain tissues form E15.5 embryos were subjected to IHC and H&E staining analyses. In all panels: CP - cortical plate; IZ - intermediate zone; VZ - ventricular zone. (**A**) illustrates the magnified views of H&E staining of littermate *BCCIP-CON* and *BCCIP-CKD* brain sections. Representative of clusters of pyknotic cell nuclei in the *BCCIP-CKD* ventricular zone are indicated by arrows. The right panels of 6A show the enlarged images of selected areas of the middle panels. (**B**) Quantification of clusters of pyknotic nuclei. (**C**) The apoptotic cells from the same were detected by TUNEL staining. (**D**) Quantification of TUNEL staining. (**E**) Proliferative cells were detected by anti-Ki67 staining (column 1), and BrdU incorporation (column 2) at E15.5 (about 2 days after the GFAP-Cre is expressed). (**F**) Quantification of Ki67 staining. (**G**) Quantification of BrdU staining. Error bars are standard deviation. White bars: BCCIP-CON; Gray bars: *BCCIP-CKD*. The asterisks indicate the statistic significance between the *BCCIP-CON* and *BCCIP-CKD*, *: P<0.05; **: P<0.01; ***: P<0.001.

To further investigate the cause of cerebrum defects, we examined *BCCIP-CON* and *BCCIP-CKD* neocortices at E15.5. As shown in Figure 6A and 6B, about two days after the activation of Cre (E15.5), there was a reduction of cell density in the cortical layer in the *BCCIP-CKD* embryos. We observed clusters of pyknotic nuclei in *BCCIP-CKD* embryos (Figure 6A and 6B). This is associated with increase of TUNEL positive cells in the same region (Figure 6C and 6D). During the same period of time, there was reduced cell proliferation capability in *BCCIP-CKD* mice based on *in vivo* bromodeoxyuridine (BrdU) incorporation assay and anti-Ki67 staining (Figure 6E). We observed much less BrdU-positive and Ki67 cells in ventricular zone (VZ) of forebrain at E15.5 than in their littermates *BCCIP-CON* (Figures 6F and 6G). These data clearly suggest a widespread proliferation defect and apoptotic activities in the progenitor cell population that later would be developed into the cerebrum structure. It further supports the essential requirement for *BCCIP* in progenitor viability and function during neurogenesis.

**Figure 7:**
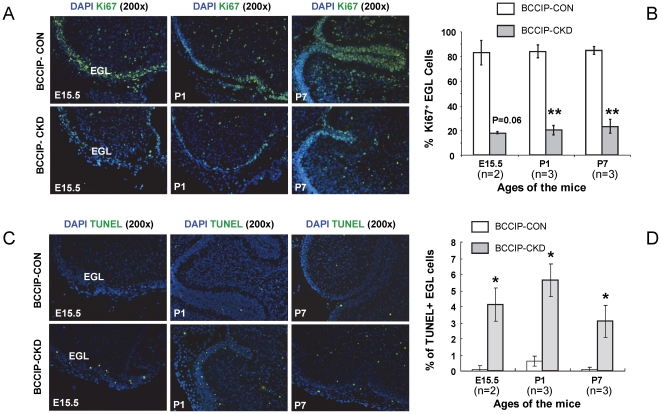
proliferation defects and excessive cell death in the external germinal layer (EGL) granule cell progenitors. Ki67 IHC staining was used to identify the proliferative cells (Green in panel A) and TUNEL assay was performed to identify the apoptotic cells (Green in panel C). DAPI staining (Blue) was used to identify the nuclei of the cells. (**A**) Ki67 staining positive proliferative cells. (**B**) Quantification of Ki67 staining. (**C**) Apoptotic cells in the EGL at age E15.5, P1, and P7. (**D**) Quantification of TUNEL staining. The “n” values indicate the pairs of littermate matched mice used in the assay. Data are averages and standard errors from the indicated number of mice. *: P<0.05; **: P<0.01; ***: P<0.001.

The mouse cerebellum development normally initiates from the dorsal region of the posterior neural tube around age E10–E12. A pool of neural progenitor cells migrates from the rhombic lip to form the external germinal layer (EGL) and give rise to the granular neuron precursor cells. Those granule neuron precursor cells are required to develop a well-structured cerebellum which undergoes over 1000-fold increase in volume during postnatal maturation [23]. We initially investigated the proliferation and cell death status at day E15.5, when the EGL structures can be readily identified. At this time, there was significantly reduced cell proliferation based on anti- Ki67 staining in EGL (Figures 7A–B), while there were significant increases of apoptotic cells in EGL (Figures 7C–D). These changes can also be observed postnatal at age P1 and P7 (Figures 7A–B). The reduced proliferation and increased apoptosis were more evident in the EGL of the cerebellum corresponding to the proliferating granule neuron progenitors.

### *In vitro* cell proliferation and self-renewal defects in BCCIP deficient neural progenitors

**Figure 8:**
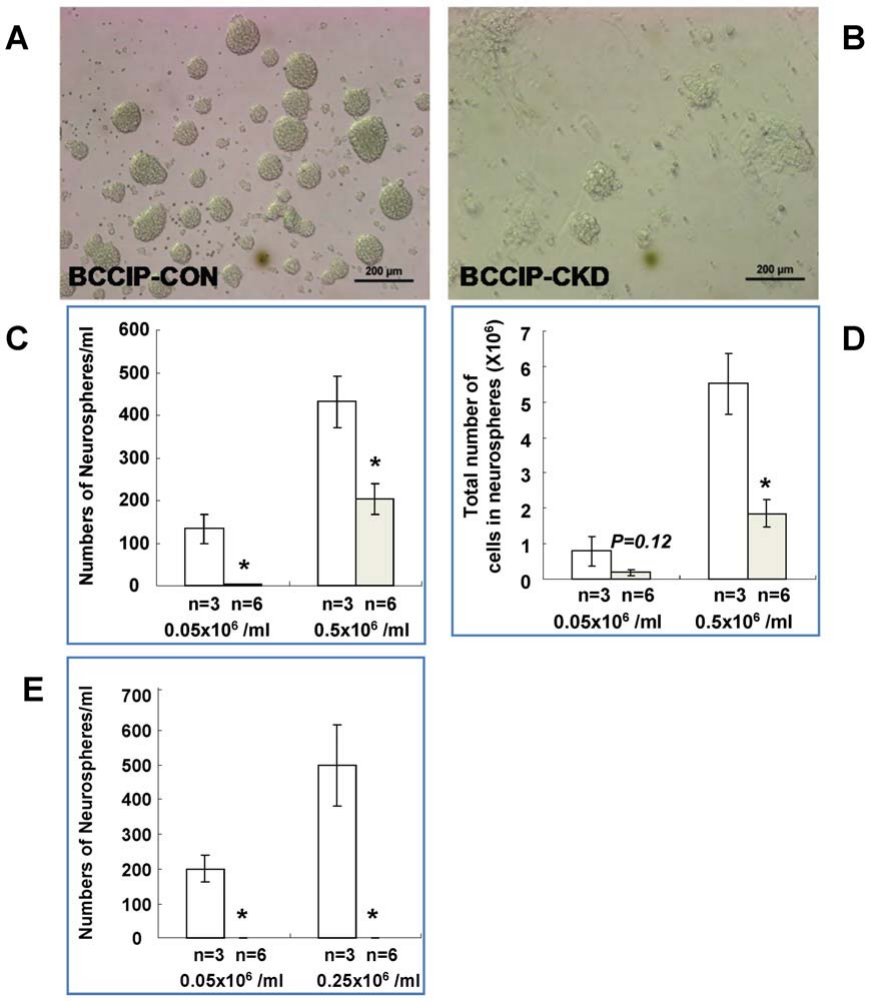
BCCIP knockdown leads to neural progenitor cell proliferation and self-renewal defects. Neural stem and progenitor cells from E15.5 brains of BCCIP-CON and BCCIP-CKD were isolated and cultured in serum-free media to allow neurosphere formation at two seeding concentrations (0.05×10^6^ cells/ml or 0.25×10^6^ cells/ml) . After 7 days in culture, and the cells were counted and re-plated to assess self-renewal ability by culturing for another 7 days. (**A**) and (**B**) shows the morphology of neurospheres originated from E15.5 BCCIP-CON and *BCCIP-CKD* mice. Scale bar = 200 mm.(**C**) shows the number of neurospheres and (**D**) shows the total cell numbers grown from the primary culture, panel (**E**) shows the number of neurospheres formed from re-suspended primary neurospheres to assess the self-renewal capability. The initial concentrations of cells plated are indicated in the figures. *: P<0.05; **: P<0.01; ***: P<0.001. Asterisks indicate significant differences and n indicates the number of individual neuroprogenitor cell lines analyzed.

**Figure 9:**
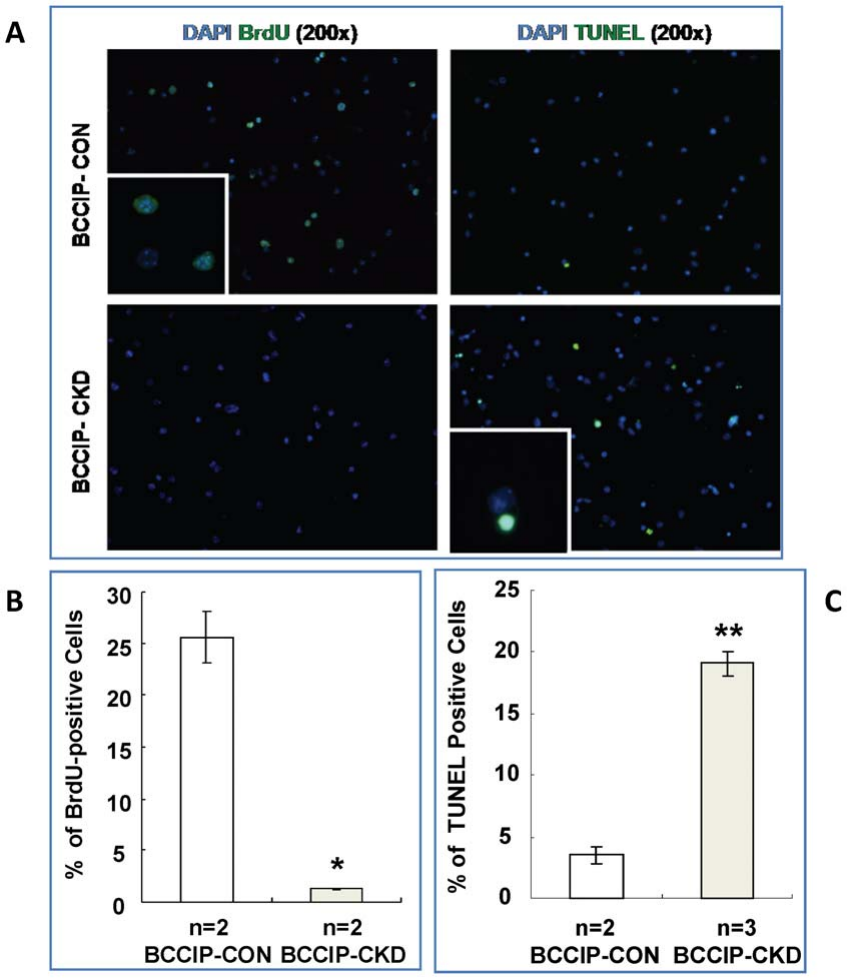
BCCIP knockdown leads to reduced BrdU incorporation and increased apoptosis in neural progenitor cells. BrdU incorporation and TUNEL assays on single cell suspension of the primary spheroid cultures were performed to assess the proliferation and apoptosis (see Materials and Methods for details). (**A**) shows representative images of BrdU and TUNEL staining. (**C**) and (**B**) show the quantification of BrdU staining and TUNEL staining. We scored 500 cells for each cell line after 7 days culture. In all panels, white bars: *BCCIP-CON*; gray bars: *BCCIP-CKD*. *: P<0.05; **: P<0.01; ***: P<0.001. Asterisks indicate significant differences and n indicates the number of individual neuroprogenitor cell lines analyzed.

The previous data (Figures 5, 6, 7) suggest that BCCIP knockdown causes proliferation defects and massive apoptotic cell death in the progenitor cell population early in embryogenesis. To confirm that BCCIP defect impairs the proliferation of neural progenitor cells, we generated neurosphere cultures from *BCCIP-CKD* and *BCCIP-CON* mice. We plated the dissociated neural cells derived from E15.5 mice (see Material and Methods). After 7 days, cultures from *BCCIP- CKD* brains formed a significantly lower number of neurospheres and of smaller size than those of *BCCIP-CON* mice (Figures 8A–8C). Accordingly, the total number of cells grown in neurospheres was significantly lower in *BCCIP-CKD* mice than B*CCIP-CON* mice (Figure 8D). To access the self-renewal capacity from primary neurospheres, we re-dissociated the collected spheroids from the primary culture, re-plated in fresh medium, and cultured for additional 7 days. As shown in Figure 8E, the re-suspended cells from *BCCIP-CON* primary neurospheres retained their ability to form secondary spheroids, but the cells from the *BCCIP-CKD* mice failed to do so. This suggests that although *BCCIP-CKD* neuron progenitor cells formed a few viable neuropsheroids, these cells had reduced ability to maintain the self-renewal potential. We further evaluated DNA synthesis by BrdU-labeling and apoptosis by TUNEL assay (Figure 9A). BCCIP knockdown decreased cell proliferation and increased the percentage of apoptotic cells (Figures 9A–9C). These data suggest that lack of BCCIP significantly impairs the proliferation of neural progenitor cells. Consistent with the *in situ* observations (Figures 5, 6, 7), they suggest that BCCIP function is essential for proliferative cells.

### BCCIP knockdown results in DNA-damage induced p53-dependent apoptosis

**Figure 10:**
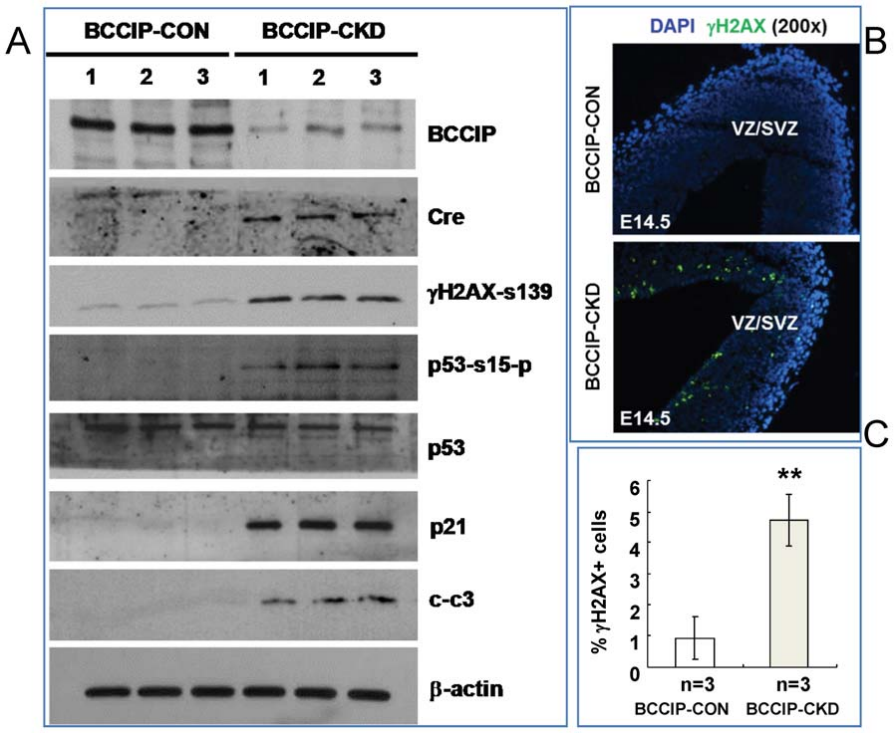
BCCIP down-regulation shows activation of phosphorylation of p53 at ser15. (**A**) Western blot from E15.5 neurospheres showed down-regulation of BCCIP expression with CRE expression. Three independent cell populations of *BCCIP-CON* and *BCCIP-CKD* were indicated on the top. Higher level of phosphorylation of H2AX (γH2AX) was shown in *BCCIP-CKD* neurospheres. All *BCCIP-CKD* neurospheres showed higher level of phosphorylation of p53 at Ser15 and p21 (a p53 downstream effector). Apoptosis was also detected of *BCCIP-CKD* mice by active caspase 3 (cleaved-caspase 3) blotting. (**B**) Immunostaining analysis of γH2AX in E14.5 *BCCIP-CKD* and *BCCIP-CON* embryos. Insets show higher magnification of γH2AX staining (magnification: 400). (**C**) Quantification of γH2AX staining. The amount of γH2AX was quantified in the VZ/SVZ of E14.5 embryos. CP: cortical plate. VZ/SVZ: ventricular zone/subventricular zone. White bars: *BCCIP-CON*; Gray bars: *BCCIP-CKD*. CP: cortical plate. VZ/SVZ: ventricular zone/subventricular zone. *: P<0.05; **: P<0.01; ***: P<0.001.

To further define the mechanisms by which BCCIP deficiency impairs progenitor cell proliferation, we analyzed the protein extracts from the *in vitro* neurospheres of *BCCIP-CON* and *BCCIP-CKD* mice (Figure 10). We found a higher level of serine-15 phosphorylation on p53 proteins in *BCCIP-CKD* neurospheres than that of the B*CCIP-CON* mice. In contrast, the total level of p53 protein was unaffected after BCCIP knockdown. The downstream effector of p53, p21 that mediates the p53-dependent cell cycle G_1_ phase arrest, was significantly increased in *BCCIP-CKD* neurospheres (Figure 10A). We also observed an elevated γH2AX level on immunoblots from neurosphere extract (Figure 10A), and a higher frequency of γH2AX-positive staining of the VZ and SVZ *in situ* (Figures 10B and 10C). These data further suggest that BCCIP deficiency causes spontaneous accumulation of DNA damage in the proliferative progenitor cells, which may trigger the activation of p53 and expression of p21 to impair proliferation of the progenitor cells.

## Discussion

Our data revealed a critical role of BCCIP in both cerebrum and cerebellum development (Figure 3). Because a reduction in proliferation was found in the VZ and EGL but not in the regions of postmitotic neurons of *BCCIP-CKD* mice (Figure 5), and an increase of apoptosis was detected in highly proliferating regions of the neocortices and EGL of *BCCIP-CKD* mice (Figures 5C, 6, 7), we suggest that BCCIP plays its roles in neural-development by supporting proliferation of neural progenitors. This conclusion is further supported by the *in vitro* neural progenitor spheroid culture studies (Figure 8), and the fact that the γH2AX foci were predominantly found in the proliferative VZ of neocortices in BCCIP deficient mice (Figure 10).

Although defective DNA damage response leads to general neurogenesis disorders, due to the preferential involvement of the each repair pathway in distinct cell population and developmental stages, deficiency on a particular DNA repair pathway often causes unique consequences [1]–[3]. Early in development and among the progenitor cell population, when cell proliferation is critical, the HR and replication related mechanisms are crucial to ensure replication fidelity and orderly development. It has been reported that conditional knockout of genes involved in HR, such as BRCA2 and Xrcc2, causes abnormalities predominantly in proliferative progenitor cells [3], [24]. In contrast, disruptions of genes involved in NHEJ, such as Ku70, DNA ligase IV, and Xrcc4, result in apoptosis of differentiating cells at later developmental stages [3], [25], [26]. The role of BCCIP in the proliferation of neural progenitor cells is consistent with a function of BCCIP in DNA replication and recombination.

In an earlier report, Frappart *et al* showed that conditional homozygous BRCA2 deletion in *Brca2^Nestin-cre^* mice resulted in neural development defects [24]. As a BRCA2 interacting protein, BCCIP has been shown to play a role in HR, cell cycle regulation, and chromosome stability [8], [11], [14], [16]. Although certain features, such as viable mice with no tumor formation, are common to conditional deletion of BRCA2 or BCCIP knockdown in neural cells, some features of the *BCCIP-CKD* mice are distinguishable from the BRCA2 knockout mice. It was noted that while the body weight of the *BCCIP-CKD* mice catches up with the littermate *BCCIP-CON* controls in adulthood, the brain size of the *BCCIP-CKD* mice remained to be at ∼50% of the controls (Figure 1F). The severity of the microcephaly is distinct from BRCA2 homozygous deletion mice, where a milder microcephaly phenotype was observed. In addition, severe ataxia was not reported in the BRCA2 deficient mice, while *BCCIP-CKD* mice displayed severe ataxia. Thus, it seems that BCCIP deficiency has a more profound effect on neurogenesis than BRCA2 deficiency.

In BRCA2, XRCC1 and NBS1 brain conditional knockout mouse models using *Nestin-Cre* [24], [27], [28], the Nestin promoter becomes active at around embryonic day 11 (E11), primarily in the central and peripheral nervous system during embryogenesis [29]. In our study, we crossed FVB-*LoxPshBCCIP^+/+^* mice with *FVB-Tg(GFAP-Cre)* transgenic mice that expresses Cre recombinase under the control of the human GFAP promoter. It is known that the onset of GFAP-mediated transgene expression occurs in the dorsal and medial regions of the telencephalon around embryonic day 13.5 (E13.5) [20]. Although only glial cells are immune-reactive for GFAP in adult brain, embryonic GFAP-promoter activity is not restricted to glial progenitor cells. Much like *Nestin-Cre*, GFAP-promoter is active in multi-potential stem cells including neuron progenitor cells during embryogenesis [20]. However, there is a difference between the two transgenic mouse strains. *Nestin-Cre* mice express Cre recombinase in common neural progenitors mostly during embryogenesis, while *GFAP-Cre* transgenic mice express Cre recombinase in both embryonic common progenitor cells as well as in adult glial cells. Thus, development defects observed in our mouse model may be attributed by defects of both neuron-progenitors and glial progenitors during the embryogenesis.

**Figure 11:**
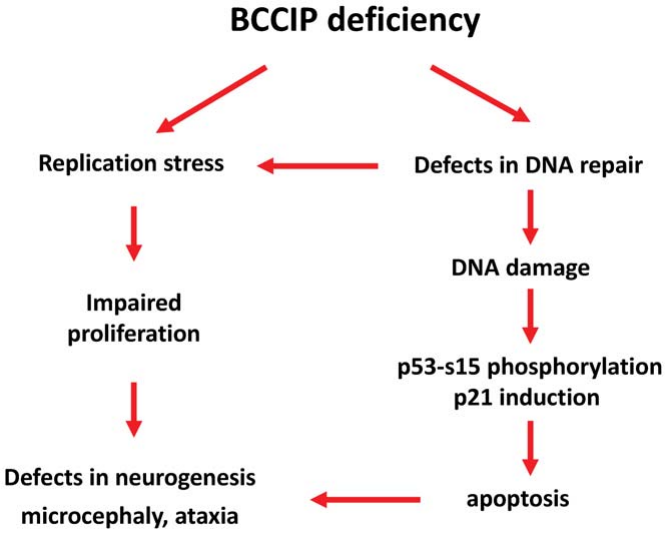
Mechanisms by which BCCIP deficiency lead to proliferation defect of the progenitor cells during brain development.

Deletion of p53 can rescue the neurogenesis defects conferred by BRCA2 deficiency, but this leads to rapid formation of medulloblastoma[24]. Nbs1 is another gene involved in DNA damage response. The *Nbs1^Nestin-Cre^* conditional knockout mice had severe neural degeneration, ataxia, and microcephaly, to a similar extent as our *BCCIP-CKD* mice [27]. The p53 deletion remarkably rescued the microcephaly and neural degeneration phenotype of *Nbs1^Nestin-Cre^* knockout mice [27]. Coincidentally, BCCIP deficiency resulted in activation of p53 (Figure 10), suggesting that p53 activation may be required for BCCIP deficiency induced neural development defects (Figure 11). It would be interesting to determine whether concurrent deletion of p53 in BCCIP deficient mice can rescue the neural development defects.

It is worthwhile to point out that we adapted a conditional BCCIP knockdown approach, in contrast to the conventionally used knockout approach. The knockdown approach enabled us to observe the phenotype of down-regulation of BCCIP gene rather than null BCCIP mutation. This may more naturally mimic the consequence of down-regulation of gene expression in development. Interestingly, in an independent transgenic line (*LoxPshBCCIP^+/+^-13*) that has a lower BCCIP knockdown efficiency than the *LoxPshBCCIP^+/+^-4* used in this report, a milder microcephaly phenotype can be observed (Supplement Figure S1). This suggests that there is a dosage-effect relationship between the degree of BCCIP down-regulation and the severity of neural development defects. An intriguing observation is that while the severe ataxia phenotype among the BCCIP deficient mice gradually improved when the mice grow into adults, we observed consistent microcephaly throughout the life span, and the majority of adult BCCIP deficient mice failed balance beam test. In addition, the reconstituted U6 promoter resulted from Cre-mediated recombination and the down regulation of BCCIP were detectable in adult BCCIP deficient mice. These observations imply that the BCCIP deficient mice may be able to make adjustment to the cerebellum defects resulted from embryogenesis.

In summary, our study suggests that BCCIP down-regulation causes severe neural development retardation due to proliferation defects of the neural progenitors. This defect is associated with a spontaneous activation of p53 and accumulation of spontaneous DNA damage in the progenitor cells. These data illustrate the critical roles of BCCIP in neural development and progenitor cell proliferation.

## Materials and Methods

### Ethics statement

The animal works presented in this study were approved by Institutional Animal Use and Care Committee of Robert Wood Johnson Medical School, University of Medicine and Dentistry of New Jersey. We follow our institutional guideline regarding to animal welfare issues.

### Mouse strains and PCR genotyping

The generation of FVB-*LoxPshBCCIP^+/+^* (founder line 4) has been described previously [16]. The *GFAP-Cre* transgenic mice (*FVB-Tg(GFAP-Cre)25Mes/J*) were obtained from the Jackson Laboratory (stock number: 004600). These mice were interbred to obtain *LoxPshBCCIP^+/−^;GFAP-Cre^−/−^* and *LoxPshBCCIP^+/−^;GFAP-Cre^+/−^* (referred to as *BCCIP-CON* and *BCCIP-CKD*) mice. During breeding, the *GFAP-Cre* transgene was routinely carried by the male to avoid germ-line BCCIP disruption due to spurious Cre expression in the ovary. The genotypes were identified by PCR of DNA prepared from tail snips. Primer sets used for genotyping are listed in the Table 1. Routine handling of mice was approved by and performed according to the guidelines for the institutional animal care committee. Balance beam test was conducted with a beam consisted of a piece of hardwood (1.5 cm wide×60 cm long) suspended 35 cm above bedding. The time that mice used to pass the beam was recorded as described by others [30].

**Table 1:** Primer sets used for genotyping.

Transgenes	Forward primer sequence(5′-3′)	Reverse primer sequence(5′-3′)	Product (bp)
U6-pLoxPneo-BCCIP	TCTAGAACTGGATCCGAC	TCGTATAGCATACATTATACG	235
GFAP-Cre	ACTCCTTCATAAAGCCCTCG	ATCACTCGTTGCATCGACCG	190
Recombined U6-BCCIP	TCTAGAACTGGATCCGAC	AGGCTTTTCTCCAAGGGATATT	317

### Histological and immune-histochemical (IHC) analysis

Embryonic brains were fixed in 4% paraformaldehyde for 24 hr, cryoprotected in 30% sucrose/PBS, and frozen for cryostatsection. All cryostatsections were cut at 8 µm. Postnatal brains were fixed in 10% buffered formalin for 24–48 hr before paraffin embedding. All paraffin embedding sections were cut at 5 µm. These sections were stained with hematoxylin and eosin (H&E) according to standard procedures. IHC analysis of tissue were performed by permeablizing with 0.1% Triton X-100 in PBS for 10 mins, quenching endogenous peroxides with 3% hydrogen peroxide for 10 mins, followed by blocking, primary and secondary antibody incubation. Immunoreactivity was visualized with 3,3′- diaminobenzidine (DAB) (D5637, Sigma). Positive staining appears as brown nuclear staining, whereas nuclei counterstained with hematoxylin appear as blue color. For fluorescence signals, Fluorescein isothiocyanate (FITC) or Rhodamine conjugated secondary antibodies were used. DAPI (4′,6-diamidino-2-phenylindole) (H-1200, VECTOR) staining was used for counterstaining. All cryosection immunofluorescence staining was performed after antigen retrieval by boiling in 0.01 M Citric acid buffer (pH 6.0). The following primary antibodies were used: calbindin D-28K (1∶500, C9848, Sigma), NeuN (1∶100, MAB377, Millipore), GFAP (1∶400, ab360, Abcam), Ki67 (1∶300, ab15580, Abcam), cleaved-caspase3 (1∶200, #9661, Cell Signaling), γH2AX(ser-139) (1∶200, #2577, Cell Signaling), BrdU (1∶100, B2531, Sigma), p53-Ser15 phosphorylated (1∶200, #9284, Cell Signaling), βIII-tubulin (1∶200, T8578, Sigma), and p21 (1∶100, sc-6246, Santa Cruz). Apoptosis was measured on cryosections after proteinase K treatment using DeadEnd Fluorometric TUNEL system (G3250, Promega).

### *In vivo* BrdU labeling

BrdU (B5002, Sigma) labeling was carried out by intraperitoneal injection of 50 mg/kg (in PBS) five times with a 2-hour interval. Mice were sacrificed 24 hours after injection and embryonic brains were processed for cryosectioning. Then, the cryosections were subjected to a 30-minute 2 M HCl treatment at 37°C, followed by routine IHC.

### Neurosphere cultures, *in vitro* progenitor cell proliferation and TUNEL assays

Embryonic day 15.5 brains were dissected in a serum-free culture medium (Dulbecco's modified Eagle medium DMEM/F-12 (1∶1). The isolated brain tissues were mechanically dissociated with a fire-polished pasture pipette and digested using an enzyme mix solution containing 30 U/ml papain (P4762, Sigma), 240 µg/ml D,L-cysteine (C7477, Sigma) and 400 µg/ml DNase I (D4527, Sigma) in DMEM/F-12 (Invitrogen). After 1 h incubation at 37°C, the enzyme mix solution was neutralized with an inhibitor solution: 0.1125% ovomucoid trypsin inhibitor (T9253, Sigma), 0.0525% BSA (A30075, Research Products International Corp.), 400 µg/ml DNase I (D4527, sigma) in L-15 medium (21083, Invitrogen). Primary cells were grown in DMEM/F-12 medium with B27 (17504-044, Invitrogen), 20 ng/ml of epidermal growth factor (EGF; E4127, Sigma) and 20 ng/ml basic fibroblast growth factor (bFGF; F0291, Sigma).

The neurosphere were grown in suspension for 6 days and in the presence of 30 µM BrdU (B5002, Sigma) for 24 hr. These spheres were collected and digested into single cell suspension, then plated on poly-L-lysine (P4832, Sigma) coated coverslips for 30 min at 37°C to allow neurosphere cells adhesion but not differentiation. Cells were fixed by 4% paraformaldehyde (P6148, Sigma) solution in PBS for 30 min at room temperature. The fixed cells were then processed for BrdU staining.

Single neurosphere cells were prepared and adhered to coverslips as described above. The fixed cells were then processed for TUNEL staining using DeadEnd Fluorometric TUNEL system (G3250, Promega). Briefly, the fixed cells were permeabilized by immersing the slides in PBS containing 0.2% Triton X-100 solution for 5 mins. Cells were incubated for 10 min in terminal-deoxynucleotidyl-transferase (TdT) buffer (Promega) before incubation with TdT and fluorescein-12-dUTP for 1 hr at 37°C. The coverslips were mounted with DAPI for nuclear stain. Localized green fluorescence of apoptotic cells were detected by fluorescence microscopy.

### Western blot

Western blots were performed with procedures as described previously [16]. Primary antibodies used were mBCCIP [16], Cre (1∶2000, 69050-3, Novagen), p53 (1∶2000, sc-6243, Santa Cruz), p53-Ser15 phosphorylated (1∶1000, #9284, Cell Signaling), p21 (1∶200, sc-6246, Santa Cruz), cleaved-caspase3 (1∶500, #9661, Cell Signaling) and γH2AX(ser-139) (1∶500, #2577, Cell Signaling).

### Statistic analyses

Data in the graphs are represented as Mean ± S.D. of replicate experiments, with the number of mice as indicated in the figures. IHC estimates were made on 3 sections per mouse, and the number of mice analyzed is indicated in each figure legend. Data obtained from *BCCIP-CKD* mice were compared with those from *BCCIP-CON* littermate controls using two-tailed Student's *t-*test. *P*-value is indicated in the graphs (^*^ *P*<0.05; ^**^ *P*<0.01; ^***^ *P*<0.001). The level of statistical significance was set at *P*<0.05.

## Supporting Information

Figure S1 **Conditional knockdown of mouse BCCIP in brain tissues using an independent founder line F13.** Panel **A** shows the genotyping of a representative litter of 8 mice resulting from breeding between *LoxPshBCCIP* ^+/+^ (founder F13) and *GFAP-Cre* ^+/−^. The brain tissues from a litter of four *BCCIP-CON* (lanes 1–4) and four *BCCIP-CKD* (lanes 5–8) mice at age P1 were used for DNA and protein extractions. The upper two panels are genotyping results from tail DNA for the presence of the split U6 promoter *LoxPshBCCIP* and the *GFAP-Cre* cassettes. The bottom panel is results of PCR genotying for the reconstituted U6-shBCCIP cassette using DNA from the brain tissue of mice. Five (lanes a, b, c, d, and e) PCR controls are: a: DNA from a (*LoxPshBCCIP* ^+/−^;*GFAPCre* ^−/−^) mouse derived from founder line F13. b: DNA from a (*LoxPshBCCIP* ^+/−^;*GFAP-Cre* ^+/−^) mouse derived from founder line F13. c: DNA from a *GFAPCre* mouse *d:* DNA from a wild type mouse e: water as a negative PCR control All 8 (No. 1-8) littermates contain the original split U6 cassette in their tail DNA. But only the littermates (No. 5-8) with the *GFAPCre* cassette have reconstituted U6-shBCCIP cassette in the DNA extracted from brain tissues at P1. Panel B shows the levels of mouse BCCIP and β-actin (loading control) protein levels from the same mice as panel A, based on Western blot analysis on the brain protein extracts. As shown here, there was a modest reduction of BCCIP protein level in BCCIP-CKD mice obtained from founder F13. The knockdown efficiency from F13 appears not as strong as founder line F4, which are shown in Figure 1 and the main text of the manuscript. Panel **C** shows the reduced brain size at p21 of F13-BCCIP-CKD compared with F4-BCCIP-CKD (the same images of the control and F4-BCCIP-CKD brains as in Figure 3 are used for comparison). Panel D shows the brain weight of F13-BCCIP-CON (white bar) and F13-BCCIP-CKD (gray bar) mice at various ages, ranging from day 1 (1D) to approximately 24 months. Asterisks indicate the statistic significance between *BCCIP-CON* and *BCCIP-CKD* of the same age (*: P<0.05; **: P<0.01; ***: P<0.001). The “n” values indicate the number of mice measured at the time point. D: day; W: week; and M: month.
(TIF)

Movie S1 **Ataxia of BCCIP deficient mice.** Shown in the video is the movement behavior of representative *BCCIP-CON* and *BCCIP-CKD* (tail tagged with the blue tape) littermates at age P21. As shown here, the *BCCIP-CKD* littermate has severe walk disability and balance disorders.
(MPEG)
