# Supplementary figures and images for: Requirement of Mouse BCCIP for Neural Development and Progenitor Proliferation
Source: PLoS One. 2012 Jan 24;7(1):e30638. doi: 10.1371/journal.pone.0030638 (PMC3265516; doi:10.1371/journal.pone.0030638)

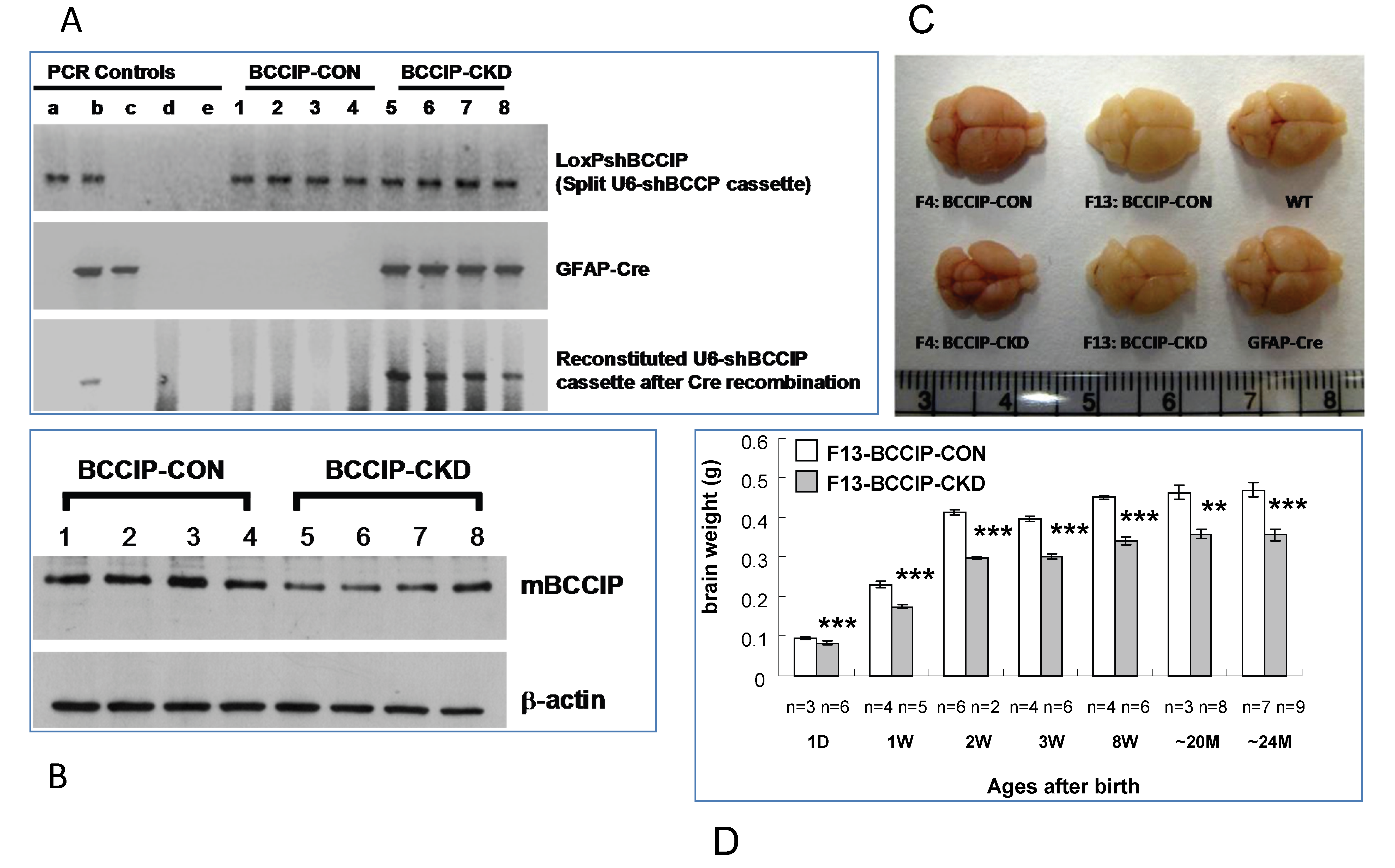

Supplement: Figure S1 — Conditional knockdown of mouse BCCIP in brain tissues using an independent founder line F13. Panel A shows the genotyping of a representative litter of 8 mice resulting from breeding between LoxPshBCCIP +/+ (founder F13) and GFAP-Cre +/−. The brain tissues from a litter of four BCCIP-CON (lanes 1–4) and four BCCIP-CKD (lanes 5–8) mice at age P1 were used for DNA and protein extractions. The upper two panels are genotyping results from tail DNA for the presence of the split U6 promoter LoxPshBCCIP and the GFAP-Cre cassettes. The bottom panel is results of PCR genotying for the reconstituted U6-shBCCIP cassette using DNA from the brain tissue of mice. Five (lanes a, b, c, d, and e) PCR controls are: a: DNA from a (LoxPshBCCIP +/−;GFAPCre −/−) mouse derived from founder line F13. b: DNA from a (LoxPshBCCIP +/−;GFAP-Cre +/−) mouse derived from founder line F13. c: DNA from a GFAPCre mouse d: DNA from a wild type mouse e: water as a negative PCR control All 8 (No. 1-8) littermates contain the original split U6 cassette in their tail DNA. But only the littermates (No. 5-8) with the GFAPCre cassette have reconstituted U6-shBCCIP cassette in the DNA extracted from brain tissues at P1. Panel B shows the levels of mouse BCCIP and β-actin (loading control) protein levels from the same mice as panel A, based on Western blot analysis on the brain protein extracts. As shown here, there was a modest reduction of BCCIP protein level in BCCIP-CKD mice obtained from founder F13. The knockdown efficiency from F13 appears not as strong as founder line F4, which are shown in Figure 1 and the main text of the manuscript. Panel C shows the reduced brain size at p21 of F13-BCCIP-CKD compared with F4-BCCIP-CKD (the same images of the control and F4-BCCIP-CKD brains as in Figure 3 are used for comparison). Panel D shows the brain weight of F13-BCCIP-CON (white bar) and F13-BCCIP-CKD (gray bar) mice at various ages, ranging from day 1 (1D) to approximately 24 months. Asterisks indicate the st [file pone.0030638.s001.tif]
